# Angiomatous meningiomas have a distinct genetic profile with multiple chromosomal polysomies including polysomy of chromosome 5

**DOI:** 10.18632/oncotarget.2517

**Published:** 2014-09-25

**Authors:** Malak S. Abedalthagafi, Parker H. Merrill, Wenya Linda Bi, Robert T. Jones, Marc L. Listewnik, Shakti H. Ramkissoon, Aaron R. Thorner, Ian F. Dunn, Rameen Beroukhim, Brian M. Alexander, Priscilla K. Brastianos, Joshua M. Francis, Rebecca D. Folkerth, Keith L. Ligon, Paul Van Hummelen, Azra H. Ligon, Sandro Santagata

**Affiliations:** ^1^ Department of Pathology, Division of Neuropathology, Brigham and Women's Hospital, Harvard Medical School, Boston, MA, USA; ^2^ Department of Neurosurgery, Brigham and Women's Hospital, Harvard Medical School, Boston, MA, USA; ^3^ Clinical Cytogenetics Laboratory, Center for Advanced Molecular Diagnostics, Department of Pathology, Brigham and Women's Hospital, Harvard Medical School, Boston, MA, USA; ^4^ Department of Medical Oncology, Dana-Farber Cancer Institute, Boston, MA, USA; ^5^ Center for Cancer Genome Discovery, Dana-Farber Cancer Institute, Boston, MA, USA; ^6^ Broad Institute of MIT and Harvard, Cambridge, MA, USA; ^7^ Department of Radiation Oncology, Brigham and Women's Hospital, Harvard Medical School, Boston, MA, USA; ^8^ Department of Neuro-Oncology, Massachusetts General Hospital, Harvard Medical School, Boston, MA, USA; ^9^ Department of Cancer Biology, Dana-Farber Cancer Institute, Boston, MA, USA

**Keywords:** meningioma, angiomatous, polysomy, aCGH, molecular diagnostics, Next Generation Sequencing

## Abstract

Meningiomas are a diverse group of tumors with a broad spectrum of histologic features. There are over 12 variants of meningioma, whose genetic features are just beginning to be described. Angiomatous meningioma is a World Health Organization (WHO) meningioma variant with a predominance of blood vessels. They are uncommon and confirming the histopathologic classification can be challenging. Given a lack of biomarkers that define the angiomatous subtype and limited understanding of the genetic changes underlying its tumorigenesis, we compared the genomic characteristics of angiomatous meningioma to more common meningioma subtypes. While typical grade I meningiomas demonstrate monosomy of chromosome 22 or lack copy number aberrations, 13 of 14 cases of angiomatous meningioma demonstrated a distinct copy number profile – polysomies of at least one chromosome, but often of many, especially in chromosomes 5, 13, and 20. WHO grade II atypical meningiomas with angiomatous features have both polysomies and genetic aberrations characteristic of other atypical meningiomas. Sequencing of over 560 cancer-relevant genes in 16 cases of angiomatous meningioma showed that these tumors lack common mutations found in other variants of meningioma. Our study demonstrates that angiomatous meningiomas have distinct genomic features that may be clinically useful for their diagnosis.

## INTRODUCTION

In adults, meningiomas are the most common primary tumor of the central nervous system [1]. These tumors arise from the leptomeninges covering the brain and the spinal cord. In the World Health Organization (WHO) Classification of Tumours, 13 histological subtypes and three grades of meningiomas are defined by histologic criteria [1]. Genetic analyses have shown that meningiomas have recurrent copy number changes associated with histologic grade. The most common aberration is monosomy of chromosome 22, with resultant loss of the *NF2* gene on 22q [2-4]. This aberration is frequently the only copy number change in WHO grade I meningiomas, with roughly 40% of grade I meningiomas displaying no recurrent changes [5]. WHO grade II and III meningiomas are associated with more complex karyotypes, most often demonstrating losses on 1p, 6q, 9p, 10q, and 14q [5]. Genomic changes in meningioma are also highly associated with histologic subtype. While inactivating mutations in *NF2* have been described in 30 to 60% of sporadic meningiomas, recent studies have shown that non-*NF2* meningiomas harbor activating mutations in *SMO* (L412F and W535L), *AKT1* (E17K), and *KLF4* (K409Q), as well as inactivating mutations of *TRAF7* [6, 7]. Notably, *SMO* and *AKT1* mutations are common in the meningothelial subtype, particularly in those arising from the base of the skull [6, 7]. Mutations in *NF2* are especially common in the fibroblastic subtype while *KLF4* and *TRAF7* are often mutated in the secretory subtype [8].

A small portion of meningiomas – approximately 2% – is classified as the angiomatous variant [9, 10]. This WHO recognized subtype is characterized by a predominance of blood vessels interspersed with small meningothelial cells and foamy spider-like cells. Most of the vascular channels have a small- to medium-sized caliber and many have vessel walls with significant thickening and hyalinization. Moderate to severe nuclear atypia is frequently present and this is ascribed to “degenerative changes” rather than being considered an indicator of malignant behavior. Moreover, meningiomas can demonstrate both angiomatous and microcystic features – the striking pleomorphism of the microcystic component potentially leading to an erroneous concern for aggressive behavior. Overall, angiomatous meningiomas do not behave aggressively [9-11]. Discriminating them from other brain tumors with hypervascularization (e.g., hemangiopericytomas and hemangioblastomas) and non-neoplastic vascular lesions (e.g., vascular malformations) is important for appropriate clinical management.

Currently, the diagnosis of an angiomatous meningioma is based exclusively on histologic review and specific molecular biomarkers supporting the diagnosis are not available. The molecular drivers of these tumors are also entirely unknown as is their genetic relationship to other vascular tumors as well as to other subtypes of meningiomas. To date, *NF2* mutations have been described very infrequently in angiomatous meningioma, suggesting that the origin of angiomatous meningiomas is largely independent of *NF2* [7, 12].

To begin to define the molecular profile of angiomatous meningiomas, we performed high-resolution array comparative genomic hybridization (aCGH) on 14 angiomatous meningiomas and exon sequencing of over 560 cancer-relevant genes in 16 of these tumors. Our work shows that angiomatous meningiomas have a distinct genetic profile characterized by numerous chromosomal polysomies and an absence of mutations commonly found in other meningioma subtypes.

## RESULTS

In 2012, the Clinical Cytogenetics Laboratory at the Brigham and Women's Hospital began characterizing all meningioma samples that were evaluated by the Neuropathology Division using a high-resolution array-based comparative genomic hybridization (aCGH) assay. This clinical test (Oncocopy) was performed in a CLIA-certified laboratory setting (see Materials and Methods). The ability of this assay to reliably detect copy number changes from DNA purified from FFPE tissues relies on a novel DNA fragmentation simulation method (FSM) [13].

**Figure 1:**
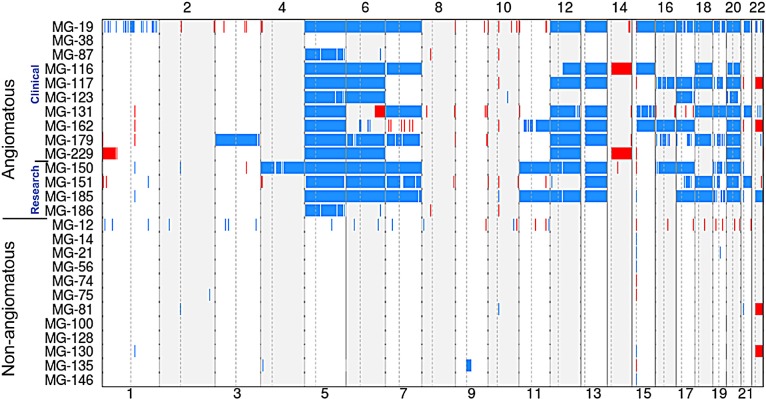
aCGH of WHO grade I meningioma and angiomatous meningioma (WHO grade I and II) Plot of copy number gains (blue) and losses (red) for 14 angiomatous meningioma samples (top) and 12 non-angiomatous WHO grade I meningiomas (bottom) that were analyzed using 1×1M Agilent SurePrint G3 Human CGH Microarray chips containing 963,029 probes with 2.1 kb overall median probe spacing and a 1.8 kb probe spacing across the human genome (Oncocopy). Cases MG-19 through MG-229 were analyzed as clinical cases in a CLIA environment by the Center for Advanced Molecular Diagnostics (CAMD) at Brigham and Women's Hospital, and MG-150 through MG-186 were analyzed as research cases, also by the CAMD. All non-angiomatous cases were analyzed for clinical diagnosis. Chromosome numbers are displayed horizontally (even numbers are listed at the top and odd numbers are listed at the bottom). Sex chromosomes were not analyzed or displayed. The segmented aCGH data was plotted using the aberration-plot function of the copy number and Bioconductor packages in R (limits −2,2).

Since the start of aCGH testing, 10 cases with a histologic diagnosis of angiomatous meningioma or meningioma with angiomatous features were evaluated by aCGH (Table S1). The assay revealed that nine of 10 tumors harbored polysomies. The whole genome profiles of somatic copy number alterations (SCNAs) are depicted in Figure 1 (blue–gain; red–loss). The nine cases with polysomy alterations were convexity meningiomas, while one case without trisomies (MG-38) originated from the petroclival region of the skull base. As is common with skull-based tumor, the MG-38 tumor sample was small and the limited tumor cells were admixed with normal dura raising the possibility that the inability to detect polysomies may have resulted from low tumor purity. We next performed aCGH analysis on four additional angiomatous meningioma cases that had been diagnosed at our hospital within the last five years and found that all four samples also harbored polysomies (Figure 1). In total, 13 of 14 meningiomas displayed polysomy of at least one chromosome.

**Figure 2:**
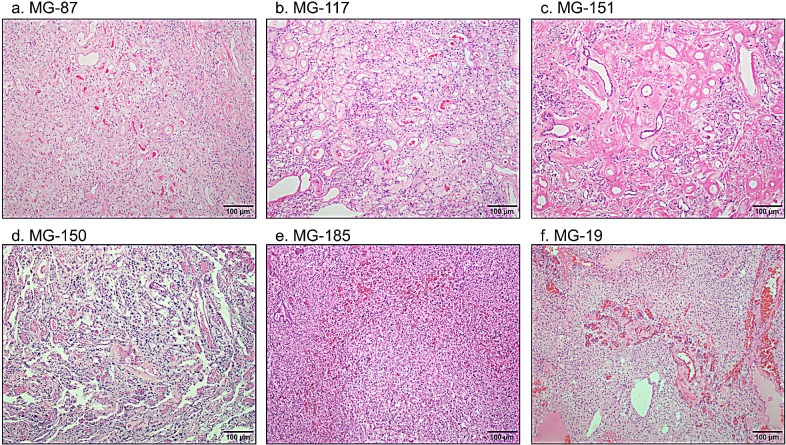
Diversity of morphologies of WHO grade I angiomatous meningioma with polysomies H&E stained sections demonstrate that while some angiomatous meningiomas have thick hyalinized blood vessels (a-c), others have a mixed profile of capillary-sized blood vessels admixed with hyalinized blood vessels (d), or only predominantly capillary-sized vessels (e, f).

**Figure 3:**
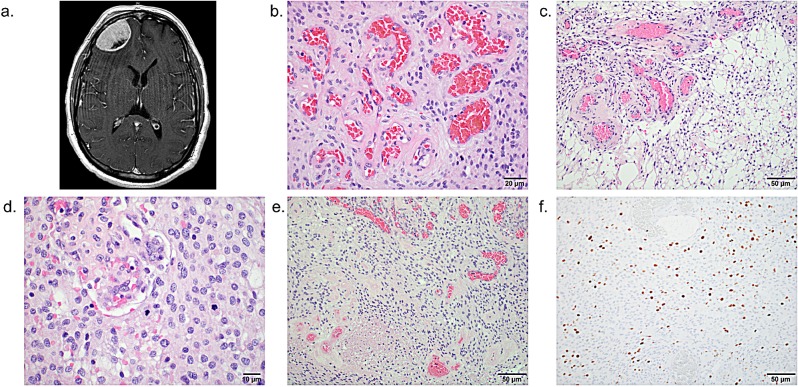
Features of a WHO grade II atypical meningioma with angiomatous features (a) Axial T1-weighted gadolinium-enhanced MRI of patient MG-229 shows a 4.6 cm right frontal convexity meningioma that extends to the orbital roof, with moderate adjacent vasogenic edema and mild effacement of the anterior horn of the right lateral ventricle. H&E stained sections reveal (b) prominent thick- and thin-walled blood vessels with (c) microcystic change, (d) numerous mitoses, (e) and foci of necrosis, and (f) immunohistochemistry shows an elevated MIB-1 proliferative index. Scale bars and measurements are shown for each image.

Of these 14 samples, 10 met WHO histopathology criteria for grade I meningioma. Some tumors demonstrated markedly hyalinized blood vessels (Figure 2a-d) while others had smaller capillary-sized blood vessels (Figure 2d-f) with MG-19 demonstrating clusters of thick-walled blood vessels separated by regions of meningothelial cells (Figure 2f). Four cases met criteria for grade II atypical meningioma with increased mitoses or other features including increased cellularity, a high nuclear to cytoplasmic ratio, sheeting, prominent nucleoli and necrosis (Table S1; e.g. MG-229 in Figure 3). For all cases, the median age was 62 years old (range 42 to 90), with six males and eight females. No cases have yet recurred, although the follow-up length is limited (less than three years for all but four of the cases).

When we compared the copy number profiles of angiomatous meningioma to a panel of 12 grade I meningiomas with subtypes that are more commonly seen in clinical practice (one fibroblastic, five meningothelial, four transitional, and two secretory), the copy number changes observed in angiomatous tumors appeared unique and striking (Figure 1). Consistent with reports in the literature, none of the fibroblastic, meningothelial, transitional, or secretory grade I cases harbored any arm-level gains and only two cases had monosomy 22 in the absence of other arm-level losses.

**Figure 4:**
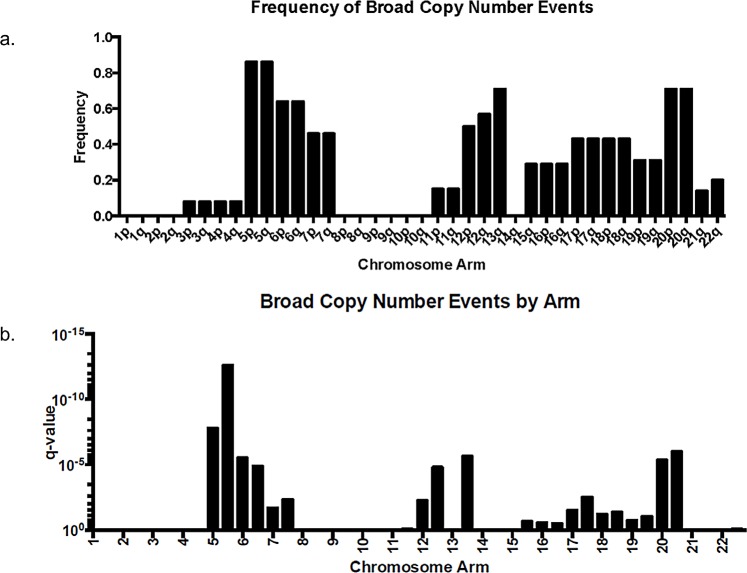
Frequency and significance of broad (arm-level) copy number aberrations in 14 angiomatous meningiomas by aCGH (a.) Frequency (% of the 14 cases having the event) and (b.) q-value data as calculated by GISTIC 2.0 [38] from the 14 angiomatous meningioma cases presented in Figure 1.

In contrast, angiomatous meningiomas had copy number gains across a range of different chromosomes. All of the 13 angiomatous cases with copy number gains had polysomy of chromosome 5 (Figures 1 and 4). In two of these cases (MG-87 and MG-186), polysomy of chromosome 5 was the only change detected – the remaining eleven cases had polysomies of at least four chromosomes, with one case (MG-19) showing wide-spread genome disruption with polysomies of 13 chromosomes (Figure 1). After chromosome 5, the chromosomes most frequently gained were 20 (11 cases) and 6, 12, and 13 (9 cases each). Polysomy 7, 17, and 18 were also present in many cases. Whole-arm gains of chromosomes 1, 2, 8, 9, 10 or 14 were not present in any of the cases (Figures 1 and 4). Loss of chromosome 3p (involving the *VHL* gene), which is frequently present in most hemangioblastoma – another highly vascular primary lesion of the central nervous system – was only observed in one angiomatous meningioma, and the pattern of other chromosomal aberrations in hemangioblastoma [14, 15] were absent in angiomatous meningioma. Hence, the copy number profiles of these two tumor types are distinct.

Four of the samples of meningioma with angiomatous features (MG-116, MG-131, MG-162, MG-229) met criteria for designation as WHO grade II atypical meningioma (MG-229 shown in Figure 3). High grade meningiomas with angiomatous features have only been very infrequently reported [9, 10, 16]. All four of the samples of atypical meningioma with angiomatous features in our cohort had multiple polysomies and three of these four samples had genomic aberrations that are typically present in WHO grade II atypical meningioma [5] (Figure S1). MG-162 lacked such changes while MG-116 demonstrated monosomy 14, MG-131 had a 47 Mb single copy loss of 6q22.31-6q27, and MG-229 had a 60 Mb single copy loss of 1p36.33-1p31.3 and monosomy 14 (Figure S1).

To determine if established cancer genes are mutated in angiomatous meningioma, we sequenced all exons from 560 cancer associated genes (Tables S1 and S2) and performed amplicon sequencing of all exons from *TRAF7* and the exon encoding the *KLF4* K409 amino acid residue that were not present in the OPv2 gene set (Table S1). Notably, mutations and deletions were not found in *NF2* in any of the 16 samples tested (Tables S3 and S4; 10 of these samples were analyzed by aCGH and six samples were not). Moreover, oncogenic driver mutations previously described in grade I meningiomas (including *AKT1*, *SMO*, *KLF4*, and *PIK3CA*) and inactivating mutations in *TRAF7* were not found in any angiomatous meningiomas. Also of note, mutations were not observed in the *VHL* gene, which is frequently mutated in hemangioblastoma [14, 15].

**Figure 5:**
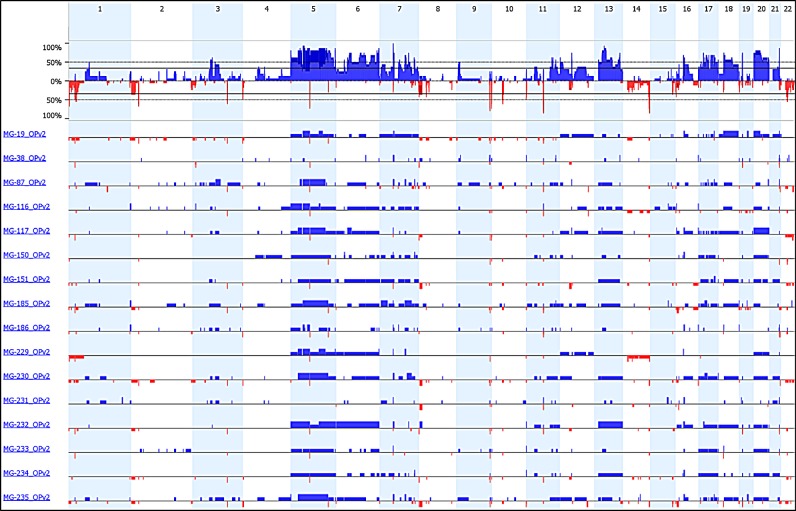
Copy number analysis from exon sequencing data of 560 cancer genes The frequency of gains and losses is shown in the upper panel. The copy number profile from each individual tumor sample is shown in the lower panel (blue – gain; red – loss).

We next performed copy number analysis from the DNA sequencing data for the 16 angiomatous meningioma cases that had undergone Oncopanel exon sequencing (Figure 5). Using this orthogonal approach, we also readily identified multiple polysomies, again with frequent gains in chromosomes 5, 13, and 20 as well as chromosomes 6, 7, 12, 17, and 18 (Figure 5). Six of these sixteen cases (Table S1) only had Oncopanel sequencing performed and of these cases, one (MG-231) did not show chromosomal polysomies. It had an unusual clustering of thin-walled blood vessels that was distinct from other tumors diagnosed as angiomatous meningioma in our cohort.

**Figure 6:**
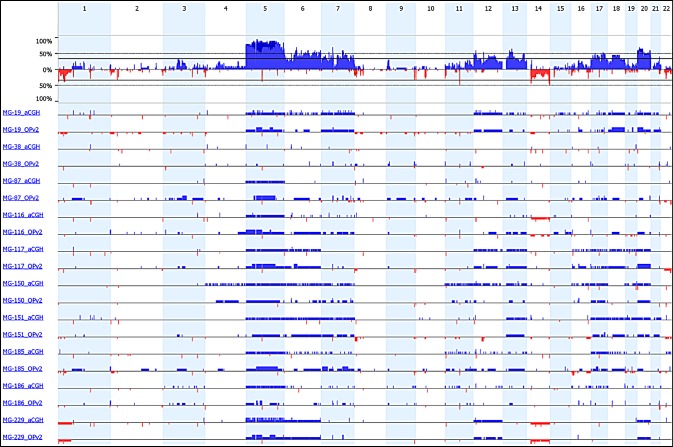
Comparison of copy number analysis from exon sequencing data and high-resolution aCGH data Ten angiomatous meningiomas were characterized by both exon sequencing (OPv2) and aCGH. The plot displays the copy number changes from the orthogonal analytical approaches (blue - gain; red - loss).

Comparing the data from 10 cases that had had both Oncopanel sequencing and aCGH Oncocopy analysis demonstrated excellent concordance (Figures 5 and 6; Table S1 and S5), indicating that the pattern of polysomies characteristic of angiomatous meningioma can be identified using exon sequencing data, as can the deletions characteristic of WHO grade II atypical meningioma (Figures 5 and 6; e.g., large deletions on chromosome 1p and 14 in MG-229). Notably, the only case (MG-38) that did not have polysomies detected by aCGH analysis also lacked polysomies in the analysis of the Oncopanel exon sequencing data (Figures 5 and 6). One apparent discrepant call is the lack of monosomy 22 in MG-117_aCGH, which is present in MG-117_OPv2. A review of the data shows that there is monosomy 22 at a fraction that does not meet the threshold called by the Nexus analysis software (Figure S2). This lesion did meet the threshold set in our prior analysis (Figure 1). Overall, using aCGH Oncocopy data as the reference point (i.e., “gold” standard), the sensitivity for identifying whole-arm copy number gains from the Oncopanel data was 85% with a specificity of 86%, and the sensitivity for identifying whole-arm copy number losses was 100% with a specificity of 93% (Table S5). This high concordance was possible even though probe spacing and density is much lower for the Oncopanel sequencing assay (Figure S3).

## DISCUSSION

Meningiomas are a heterogeneous group of tumors – by both histopathology and molecular profiling. To date, angiomatous meningiomas have been recognized as a WHO grade I subtype, however, there have been no specific molecular aberrations ascribed to this histologic subclass. Our study demonstrates that angiomatous meningiomas are distinct from other meningiomas – bearing numerous chromosomal polysomies and lacking mutations characteristic of other meningioma subtypes. These polysomies can be detected using aCGH analysis, as is now standard in our molecular diagnostics laboratory using our Oncocopy assay, or by exon sequencing strategies using a panel of genes such as Oncopanel [17]. These findings can help diagnostic classification and may shed light on the molecular pathogenesis that underlies the development of these tumors.

The pattern of chromosomal alterations in angiomatous meningioma is strikingly different than other grade I meningiomas, which most commonly harbor monosomy 22 or no recurrent chromosomal changes. Typically, aneuploidy is viewed as imposing tremendous proteotoxic stress on cells [18, 19], which can be detrimental to survival, growth and proliferation [19-22]. As such, aneuploidy and the associated cell stress render cancer cells preferentially susceptible to treatments that target the stress phenotype of malignant cells [23-25] and the aneuploidy state in particular [26, 27]. Despite the pressure placed on cells, aneuploidy has long been recognized as common feature of most cancer genomes, particularly the genomes of aggressive tumor types.

Recently, a cumulative analyses of aneuploidy across cancer genomes has shown that the distribution and potency of tumor suppressor genes (termed “STOP” genes), as well as oncogene and essential genes (termed “GO” genes) on chromosomes are responsible for the recurrent patterns of aneuploidy and copy number variation that are observed in cancer [28, 29]. Interestingly, we detected highly recurrent gains of chromosomes 5, 13, and 20 (most frequent changes) as well as gains of 6, 7, 12, 15, 16, 17, 18, and 19 (less frequent changes). Across many cancers, the chromosomes that are most frequently gained are 7, 20, 8, 13, 12, and 5 [28, 29]. Angiomatous meningiomas display gains of five of these six chromosomes (all but chromosome 8), suggesting that the net growth and proliferation signals present on these chromosomes outweighs the inhibitory growth signals and the burden of stress placed by the aneuploidy state.

Moreover, the signal for vascular differentiation, development, and proliferation – a hallmark of angiomatous meningioma – may reside on these regions of chromosomal gain. Interestingly, the VEGF pathway has been implicated in the pathogenesis of angiomatous meningiomas [30] and the *VEGF-A* gene is located on chromosome 6, which is frequently gained in these tumors. Other genes with roles in vascular development and proliferation reside on chromosome 5 and include *PDGFRB, AGGF1, FGFR4*, and *FGF10*. Notably, *TERT* is also present on chromosome 5.

Mutations in the *TERT* promoter and increased TERT mRNA expression were recently reported in a subset of meningioma that recur and undergo malignant progression [31]. Conceivably, the copy number gains on chromosome 5 in angiomatous meningioma – which do not typically demonstrate aggressive clinical behavior [9, 10] – do not elevate TERT mRNA and protein levels to the same level as promoter mutations do. The effects of elevated TERT levels on tumor progression likely also require other companion mutations or genomic alterations that are not present in angiomatous meningioma.

Despite the significant aneuploidy encountered in angiomatous meningiomas, these tumors do not generally display aggressive behavior and patients have very favorable prognoses [9, 10]. Indeed, the presence of aneuploidy is not uniformly associated with worse clinical outcome. For instance, genetic analysis has revealed that there are two distinct molecular variants of ependymomas of the posterior fossa [32]. One subgroup has a paucity of copy number aberrations and poor outcome while the other subgroup harbors multiple recurrent chromosome arm-level cytogenetic aberrations including losses of chromosomes 1, 2, 3, 6, 8, 10, 14q, 17q, and 22q and gains of 4, 5q, 7, 9, 11, 12, 15q, 18, 20, and 21q; yet, the latter is associated with excellent survival [32-34].

Hyperdiploidy has been previously described in 2.4% of meningioma in a cohort of 677 meningiomas using spectral karyotyping techniques [35]. Ten of 15 of these hyperdiploid cases were described as having microcystic features and it was mentioned that those specimens had numerous blood vessels suggesting that many of those cases may have had angiomatous features. The diagnosis of angiomatous meningioma was not discussed in that work, however, and it is unclear whether the five remaining hyperdiploid cases without microcystic features also displayed numerous blood vessels. In our cohort, six of 20 cases also had microcystic features. Taken together, both studies support a molecular overlap for these meningioma subtypes which frequently co-exist. The prior work with karyotyping suggests that a subset of meningioma with chromosomal polysomies may not display angiomatous features. Further analysis and independent studies will be needed to assess the sensitivity and specificity of chromosomal polysomies in the diagnosis of angiomatous meningioma.

While angiomatous meningiomas are not generally clinically aggressive tumors, four cases in our cohort had atypical features suggesting that a subset of these tumors might have a tendency to recur. Future insights into the molecular genetic drivers of these tumors that build upon the molecular analysis presented in this work might inform our understanding of the pathobiology of these tumors and of possible new therapeutic strategies.

## MATERIALS AND METHODS

### Specimens and clinical characteristics

Research aCGH testing on human tumor specimens and analysis of clinical aCGH data was conducted following the approval from the Dana-Farber/Brigham and Women's Cancer Center (DF/BWCC) Institutional Review Board. All aCGH testing was performed within a CLIA-certified laboratory environment in the Clinical Cytogenetics Laboratory/Center for Advanced Molecular Diagnostics (CAMD) at Brigham and Women's Hospital. All tumor samples were reviewed by at least two neuropathologists (M.A.A. and S.S.) and were classified according to the World Health Organization (WHO) criteria of brain tumors. Fourteen angiomatous meningioma samples were analyzed by aCGH, of which 10 were processed as clinical specimens and four as research specimens. A group of 12 WHO grade I meningioma including meningothelial, fibrous, transitional, and secretory subtypes that had undergone clinical aCGH testing were analyzed as a comparative group. This group had a similar age and gender distribution as the group of angiomatous meningioma. Targeted sequencing of 560 cancer-related genes was performed on 16 angiomatous meningiomas and amplicon sequencing of coding exons from *TRAF7* and for recurrent mutation in *KLF4^K409Q^* was performed on 15 cases (Table S1). Ten angiomatous meningioma had both aCGH and sequencing analysis (Table S1). The MIB-1 proliferation index was taken from the clinical report.

### aCGH using FFPE samples

To identify tumor-specific genomic copy number alterations, we performed Oncocopy, an array-based comparative genomic hybridization (aCGH) test using a stock 1×1M Agilent SurePrint G3 Human CGH Microarray chip. A minimum of 1.3 μg of DNA, corresponding to approximately 10 × 5 μm standard FFPE sections or 6 × 1 mm punches containing at least 50% tumor, was obtained for each specimen. Genomic DNA isolated from FFPE blocks was hybridized with genomic DNA isolated from a commercial reference DNA sample representing a pool of individuals with normal karyotypes (Promega, Madison, WI). The array platform contains 963,029 probes spaced with a 2.1 kb overall median probe spacing and a 1.8 kb probe spacing in RefSeq genes across the human genome. Data analysis was performed according to standard settings of the CLIA laboratory.

### aCGH analysis

Raw copy number data in the form of aCGH text files were mapped using *Homo sapiens* UCSC hg18 build. Array normalization and removal of unmapped probes were conducted using algorithms provided by the Memorial Sloan Kettering Cancer Center, Cancer Genome Characterization Center (Novel normalization algorithms and QA measure for array CGH, (http://cbio.mskcc.org/CGCC/)

Circular Binary Segmentation (CBS) was used to segment copy-number data from research aCGH [36, 37]. CBS was performed using Matlab (The MathWorks, Inc., Natick, MA) with parameters of α = 0.01, undo.splits = none, minimum width = 5. Segmented data were analyzed using GISTIC 2.0 [38] to determine statistically significant recurrent copy number alterations (CNAs), after filtering germline copy number variations, using the following parameters: minimum segment size of 10, focal versus broad CNA events defined with a cutoff of 0.5x chromosome arm length, and gene confidence level of 0.99. Copy number alterations were called when associated with log2 copy number changes >0.2. Analysis of significant CNAs excluded chromosomes X and Y [38]. Segmented aCGH data and Oncopanel Data were plotted using the aberration-plot function of the copy number and Bioconductor packages in R [39, 40]. GISTIC CNAs were plotted using GraphPad Prism (GraphPad Software, San Diego, CA).

### DNA isolation and exon sequencing of angiomatous meningioma FFPE samples

We obtained DNA from approximately 5-10 × 0.6 mm punches (diameter 1 mm, Miltex, Plainsboro, NJ) from formalin-fixed paraffin-embedded (FFPE) tissue blocks containing at least 50% tumor using QIAamp DNA FFPE Tissue Kit (Qiagen, Valencia, CA). The concentration of double-stranded DNA was quantified using Quant-iT™ PicoGreen® dsDNA Assay Kit (Life Technologies, Carlsbad, CA). Tumor samples were resected between 2003 and 2014.

Exons of 560 cancer-relevant genes were sequenced at the Center for Cancer Genome Discovery (CCGD) at the Dana-Farber Cancer Institute (OncoPanel version 2, OPv2), using an Illumina sequencing platform. Prior to preparation of the library, 200 ng of double-stranded DNA was fragmented to approximately 250bp segments using Covaris sonication (LE220 Focused-ultrasonicator, Covaris, Woburn, MA). Fragmented DNA was purified using Agencourt AMPure XP beads (Beckman Coulter, Inc. Indianapolis, IN). A total of 50 ng of size-selected DNA was then ligated to sequencing adaptors with sample-specific barcodes (Illumina TruSeq) and quantified by qPCR. For exon enrichment, libraries were pooled in equal mass to a total of 500 ng, and exonic regions were captured with the SureSelect Target Enrichment system (Agilent Technologies, Santa Clara, CA). The capture pool was sequenced in one lane of the HiSeq 2500 system (Illumina Inc., San Diego, CA) in Rapid Run Mode.

We de-multiplexed the pooled sample reads and sorted the data using Picard tools (http://picard.sourceforge.net/command-line-overview.shtml). We aligned the reads to the reference sequence b37 edition from the Human Genome Reference Consortium, using bwa (http://bio-bwa.sourceforge.net/bwa.shtml) – parameters “-q 5 -l 32 -k 2 -o 1.” Duplicate reads were removed using the Picard tools [41]. The alignments were further refined using the GATK tool for localized realignment around indel sites (http://www.broadinstitute.org/gsa/wiki/index.php/Local_realignment_around_indels).

Recalibration of the quality scores was also performed using GATK tools (http://www.broadinstitute.org/gsa/wiki/index.php/Base_quality_score_recalibration) [42, 43]. The mean target coverage across all samples was on average 102x with 89% of all targets at 30x coverage or more.

### Oncopanel variant analysis

Mutation analysis for single nucleotide variants (SNV) was performed using MuTect v1.1.4 [44] and annotated by Oncotator (http://www.broadinstitute.org/oncotator/). We used the SomaticIndelDetector tool that is part of the GATK for indel calling. MuTect and SomaticIndelDetector were run in either paired mode using a matched normal as the germline filter, or in paired mode using internal control CEPH as the normal for tumor samples without a match. A germline variant filter was applied to the tumors with no matched normals. Non-synonymous variants were filtered against the 6,500 exome release of the Exome Sequencing Project (ESP) database. Variants represented at >1% in either the African-American or European-American and not in COSMIC > 2x were considered to be germline. There were very few variants in the tumors with matched normal. In the tumors without matched normal mutations were generally 10-20 times higher. Listed in Table S2 are the 560 sequenced genes and the 39 chromosomal breakpoints that were evaluated.

### Oncopanel copy number analysis

We performed copy number analysis using Nexus7.1 (BioDiscovery Inc.) after calculating the sequencing coverage using PICARD. Coverages were normalized over GC-content using a lowest regression and a CCGD normal DNA (17_L_000344) as reference. CNVs were called using next generation sequencing (NGS) versus aCGH settings presented in Table S5.

### Amplicon sequencing of TRAF7 and KLF4

Targeted exon enrichment was performed to assess the mutational status of *TRAF7* and *KLF4* using a custom Qiagen GeneRead DNAseq panel following the manufacturer's suggested protocol (Qiagen, Valencia, CA). Each sample was prepared and sequenced in duplicate to identify and eliminate PCR artifacts. Sequencing libraries were prepared using the NEBNext DNA Library Prep methods and barcoded with unique indices. Libraries were pooled in equimolar concentrations and sequenced on an Illumina MiSeq using150bp paired end reads to an average depth of approximately 4,500x. Data analysis was performed using the Qiagen NGS Data Analysis Web Portal. All alterations were manually inspected by Integrated Genome Viewer (IGV) (http://www.broadinstitute.org/igv).

## SUPPLEMENTARY MATERIAL FIGURES AND TABLES




