# Supplementary material for: Angiomatous meningiomas have a distinct genetic profile with multiple chromosomal polysomies including polysomy of chromosome 5
Source: Oncotarget. 2014 Sep 25;5(21):10596–606. doi: 10.18632/oncotarget.2517 (PMC4279396; doi:10.18632/oncotarget.2517)
Supplement: Supplementary file 1 [file oncotarget-05-10596-s001.pdf]

Angiomatous meningiomas have a distinct genetic profile with multiple chromosomal polysomies including polysomy of chromosome 5

Supplementary Material

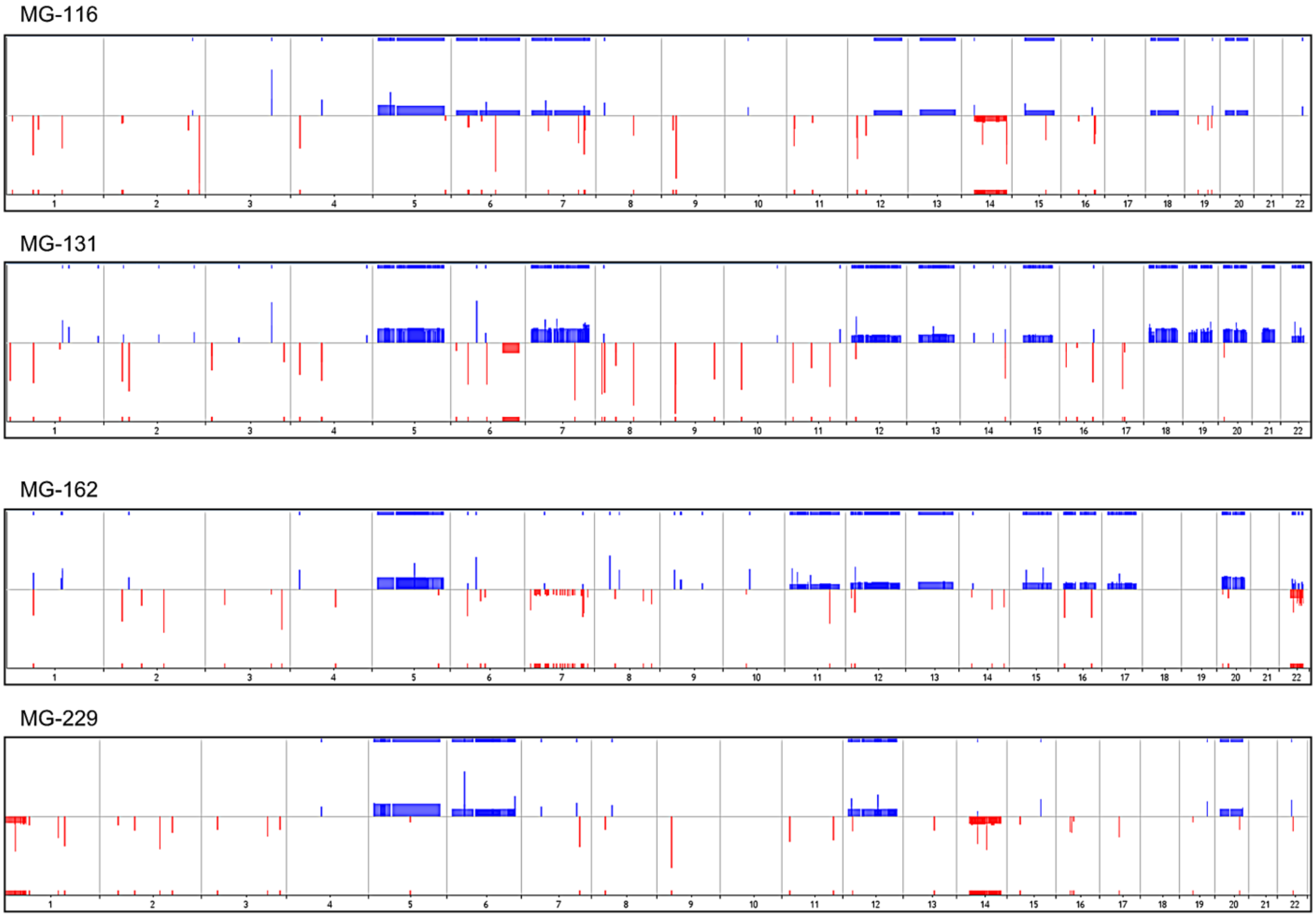

Figure S1: Copy number alterations in a WHO grade II atypical meningioma with angiomatous features (MG-116, MG-131, MG-162, MG-229). Plots of copy number alterations with Agilent Cytogenomics Edition 2.7.8.0 software (blue–gain; red–loss) showing polysomies as well as genomic aberrations consistent with grade II atypical meningioma (monosomy 14 in MG-116, a loss on 6q in MG-131, a loss on 1p and monosomy 14 in MG-229). Monosomy 22 is present in MG-162. Sex chromosomes were not analyzed or displayed.

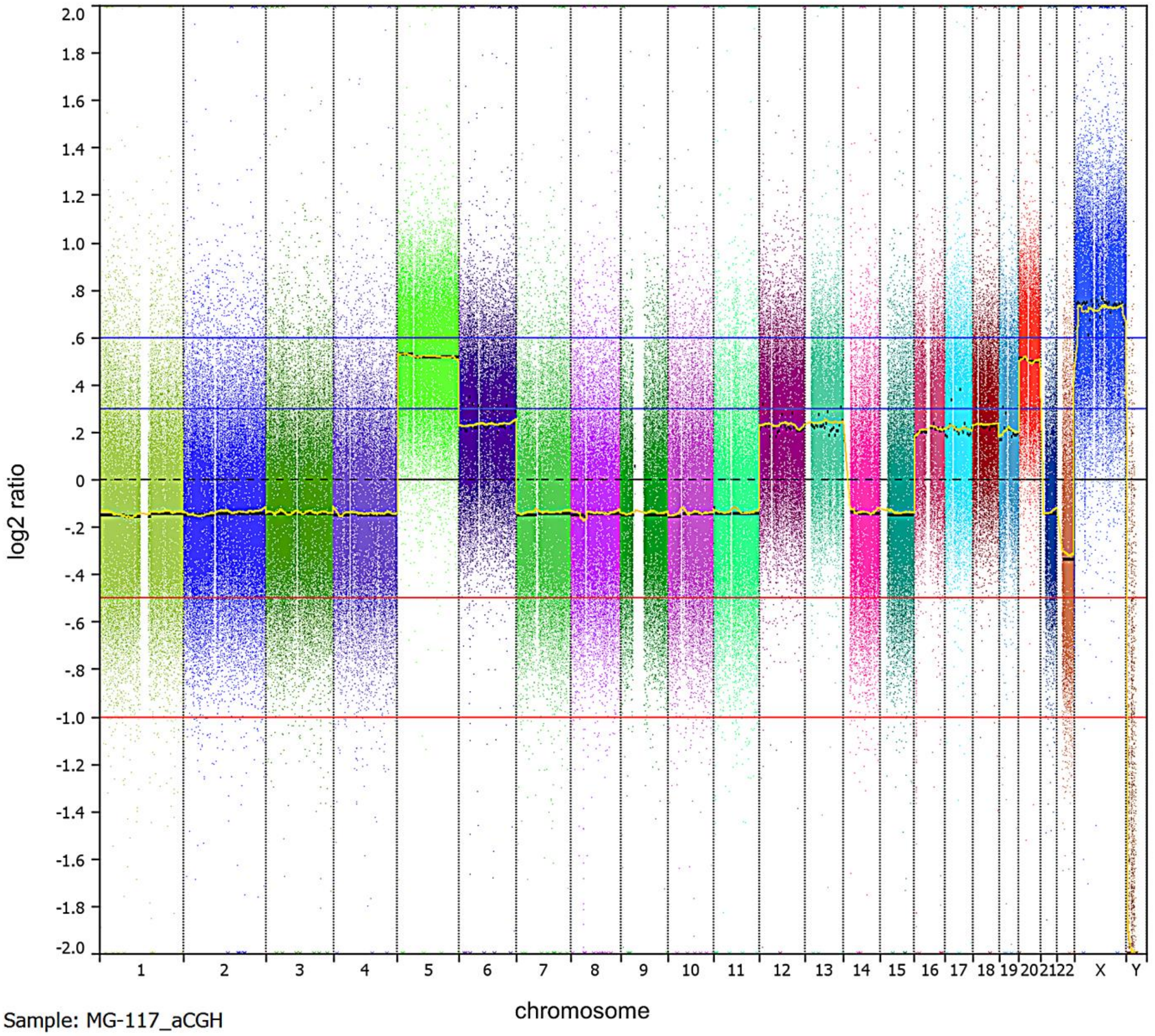

Figure S2: Plot of signal from aCGH probes for MG-117 showing a sub-threshold level of monosomy 22. Log2 ratios are shown. The plot was generated by Nexus analysis software. The moving average is indicated by the black line. Because of the extensive aneuploidy, the median value of all probes for this sample is between 0 and -0.2.

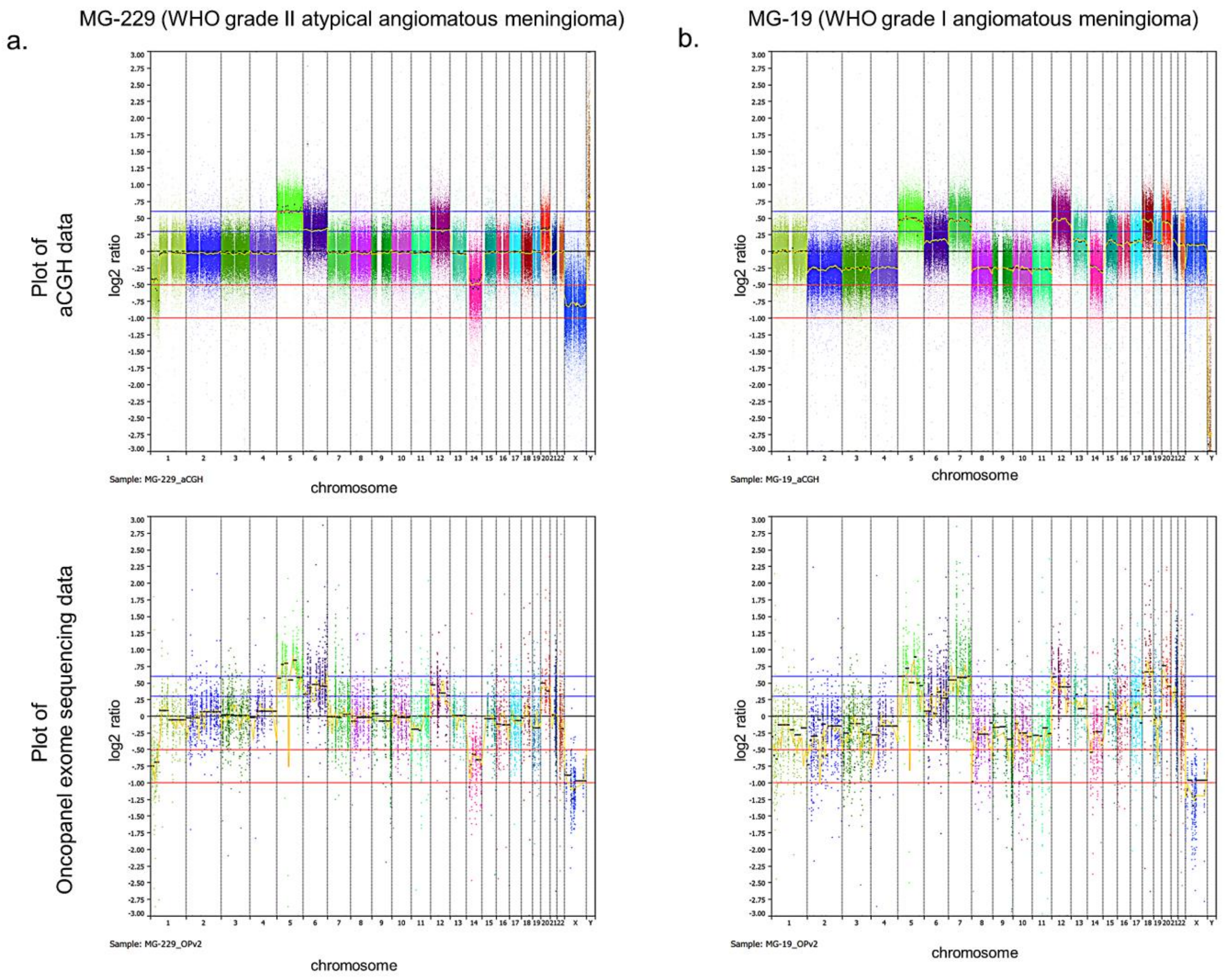

Figure S3: Plots of signal from aCGH probes and Oncopanel exon sequencing probes. The top panels display the signal from each probe from the high-density aCGH arrays while the bottom panels display the signal from each probe from the more sparsely covered Oncopanel exon sequencing. Log2 ratios are shown. Plots generated by Nexus analysis software. (a) MG-229 is the WHO grade II atypical meningioma with angiomatous features (shown in Figure 3) and displays numerous polysomies as well as losses on chromosome 1p and monosomy 14 by both aCGH (upper panel) and Oncopanel (lower panel). (b) MG-19 is a WHO grade I angiomatous meningioma and displays numerous polysomies. The moving average is indicated by the black line. Because of the extensive aneuploidy, the median value of all probes for this sample is between 0 and -0.2.
